# In Vitro Phenotypic Screening and MALDI-TOF Identification of Lactic Acid Bacteria Isolated from Feces of Suckling Piglets with Antibacterial Activity

**DOI:** 10.3390/ani16101426

**Published:** 2026-05-07

**Authors:** Nattakarn Awaiwanont, Montira Intanon, Duangporn Pichpol, Panuwat Yamsakul

**Affiliations:** School of Veterinary Medicine, Faculty of Veterinary Medicine, Chiang Mai University, Chiang Mai 50100, Chiang Mai Province, Thailand; nattakarn.a@cmu.ac.th (N.A.); montira.intanon@cmu.ac.th (M.I.); duangporn.p@cmu.ac.th (D.P.)

**Keywords:** piglets, lactic acid bacteria, in vitro screening, MALDI-TOF, antibacterial activity

## Abstract

Lactic acid bacteria are important components of the early gut microbiota in piglets and may contribute to gastrointestinal stability and control of enteric pathogens, particularly under increasing restrictions on antibiotic use in livestock production. This study aimed to perform in vitro phenotypic screening and characterization of lactic acid bacteria isolated from feces of suckling piglets aged 7–28 days. A total of 42 fecal samples were collected and cultured on selective media, yielding 318 colonies, of which 135 Gram-positive, rod-shaped, catalase-negative isolates were selected for further evaluation. These isolates were assessed for tolerance to acidic conditions (pH 2.0–3.1), bile salts (0.3–0.5%), cell surface hydrophobicity, hemolytic activity, and antibacterial activity against *Escherichia coli* and *Staphylococcus aureus*. Among the tested isolates, only two (PMvet212 and PMvet318) demonstrated limited tolerance to acidic and bile conditions and exhibited moderate antibacterial activity, with inhibition zones of approximately 10–12 mm. Identification using matrix-assisted laser desorption/ionization time-of-flight (MALDI-TOF) mass spectrometry indicated that PMvet212 was closely related to *Lactobacillus brevis*, whereas PMvet318 was identified at the genus level as *Lactobacillus* sp. However, both isolates exhibited α-hemolytic activity and therefore did not meet the safety criteria required for probiotic application. In conclusion, although piglet-derived lactic acid bacteria may exhibit certain functional properties, the present findings highlight that probiotic potential is strain-specific and that rigorous multi-step screening, together with further in vivo validation, is essential before practical application can be considered.

## Simple Summary

Probiotics are increasingly used as alternatives to antibiotics in livestock production, but identifying suitable strains remains challenging. In this study, bacteria were isolated from suckling piglets and evaluated through a stepwise screening process, including tolerance to gastrointestinal conditions, safety assessment, and antimicrobial activity. Although several isolates showed partial functional properties, none fulfilled all required criteria due to safety limitations. These findings highlight the importance of applying rigorous multi-step screening procedures when selecting host-derived probiotic candidates. The results provide a practical framework for early-stage probiotic evaluation in swine and emphasize that most naturally derived isolates do not meet the minimum requirements for probiotic application without further validation.

## 1. Introduction

*Escherichia coli* (*E. coli*) is one of the most important pathogens causing diarrhea in neonatal and weaned piglets. Piglet diarrhea leads to substantial economic losses in the swine industry due to increased morbidity and mortality, reduced growth performance, and higher veterinary costs [1]. Prevention is generally more effective than treatment; however, traditional control strategies have relied heavily on antibiotics [2]. This dependence has raised serious concerns regarding antibiotic residues in animal products and the global spread of antimicrobial resistance (AMR) [3]. As regulatory authorities and consumers increasingly demand reductions in antibiotic use, there is a growing need for alternative approaches that can maintain animal health and productivity without contributing to AMR [4]. Among the available strategies, probiotics have received considerable attention due to their potential to improve intestinal health, reduce enteric infections, and support growth performance in pigs [5]. In addition, the use of probiotics is consistent with the One Health approach for mitigating AMR across animal, human, and environmental interfaces [6].

At birth, the gastrointestinal tract of piglets is rapidly colonized by microorganisms derived from the sow and the surrounding environment. This early colonization plays a critical role in the development of digestive, metabolic, and immune functions [7]. The composition of the gut microbiota is influenced by several factors, including maternal microbiota, diet, and environmental exposure [8]. In particular, the suckling period (approximately 7–28 days of age) represents a crucial stage in microbial succession, during which beneficial bacteria such as lactic acid bacteria (LAB) become established and contribute to gut stability and resistance to pathogens [9]. Therefore, isolating LAB from piglets at this early stage may provide valuable insights into host-adapted bacterial populations with potential functional relevance. Despite numerous studies reporting the isolation of lactic acid bacteria from livestock, relatively few studies explicitly describe the attrition of candidate strains during early-stage screening under combined functional and safety criteria. In practice, a large proportion of isolates fail to meet the minimum requirements for probiotic application. Therefore, the present study aims not only to isolate presumptive LAB from suckling piglets but also to systematically evaluate their survival, functional potential, and safety characteristics within a structured screening pipeline.

Probiotics are defined as live microorganisms that, when administered in adequate amounts, confer health benefits to the host (FAO/WHO, 2002) [10]. To be considered suitable, probiotic microorganisms must meet several minimum criteria, including tolerance to gastric acid and bile salts, the ability to adhere to intestinal surfaces, absence of harmful activities, and antagonistic effects against pathogens [10,11]. LAB are among the most widely studied probiotic groups because of their generally recognized as safe (GRAS) status, their ability to survive gastrointestinal conditions, and their capacity to produce antimicrobial compounds such as organic acids and bacteriocins [12]. However, these functional properties are highly strain-specific, and not all LAB isolates meet the required criteria for probiotic application.

Recent advances in microbial identification techniques, such as matrix-assisted laser desorption/ionization time-of-flight (MALDI-TOF) mass spectrometry, have enabled rapid and reliable identification of bacterial isolates at the genus and species levels [13]. This approach provides significant advantages over traditional phenotypic methods by improving the accuracy of taxonomic classification and facilitating comparisons with previously characterized strains. Incorporating such identification methods is essential for interpreting the functional relevance of LAB isolates and for aligning experimental findings with existing microbiological knowledge.

Therefore, the aim of the present study was to perform in vitro phenotypic screening and characterization of LAB isolated from fecal samples of piglets raised without routine antibiotic or probiotic supplementation. The study focused on identifying isolates that exhibited tolerance to simulated gastrointestinal conditions, antibacterial activity against *E. coli*, and selected functional properties relevant to probiotic evaluation. In addition, selected isolates were identified using MALDI-TOF mass spectrometry to provide preliminary taxonomic classification. The findings of this study contribute to the understanding of piglet-associated LAB and provide a basis for further molecular characterization and functional validation.

## 2. Materials and Methods

### 2.1. Ethical Approval

Fecal samples were collected non-invasively from freshly voided feces without handling or restraining the animals. Therefore, specific animal use approval was not required in accordance with institutional and national animal welfare guidelines. However, all procedures involving microbial isolates were reviewed and approved by the Institutional Biosafety Committee (IBC) of Chiang Mai University (Approval No. CMUIBC A-0763004).

### 2.2. Sample Collection

The study population comprised suckling piglets aged 7–28 days. A total of 42 fecal samples were collected from 10 commercial swine farms in Lamphun Province, Thailand, with samples obtained from multiple farrowing pens (approximately 1–5 samples per pen). The selected farms did not use antibiotics or probiotics routinely, except for therapeutic purposes when necessary. Freshly voided feces with normal consistency and yellow to brown coloration were collected aseptically from the pen floor, placed in sterile 5 mL microcentrifuge tubes (≥2 g per sample), and transported to the laboratory at 4 °C. All samples were processed within 6 h after collection to minimize changes in microbial composition.

### 2.3. Isolation of Lactic Acid Bacteria (LAB)

One gram of each fecal sample was homogenized in 9 mL of sterile 0.85% saline solution and serially diluted (10^−4^ to 10^−8^). Aliquots (100 µL) were spread onto de Man, Rogosa and Sharpe (MRS) agar supplemented with 0.5% CaCO_3_. Plates were incubated anaerobically at 37 °C for 48 h using an anaerobic jar system with gas-generating sachets (Oxoid Ltd., Basingstoke, Hampshire, UK). Colonies producing clear zones were selected and repeatedly streaked on MRS agar to obtain pure cultures. Gram-positive, non-spore-forming isolates were further tested for catalase activity, and only catalase-negative isolates were preserved at −20 °C in MRS broth containing 20% (*v*/*v*) sterile glycerol [14].

### 2.4. Evaluation of Probiotic Properties

#### 2.4.1. Acid Tolerance

Bacterial suspensions were prepared in phosphate-buffered saline (PBS; pH 7.2) and adjusted to a 0.5 McFarland standard (~1.5 × 10^8^ CFU/mL). One milliliter of suspension was mixed with 9 mL of PBS adjusted to pH 2.0 or pH 3.1 using 1 N HCl. The mixtures were incubated at 37 °C for 3 h. Viable cell counts before and after incubation were determined by plate counting on MRS agar. Acid tolerance was expressed as the reduction in viable counts (log CFU/mL) compared to the initial value [15]. The exposure time and pH conditions were applied as a stringent in vitro screening approach to evaluate bacterial survival under severe acid stress, rather than to directly simulate the physiological gastric transit conditions in piglets.

#### 2.4.2. Bile Salt Tolerance

Bacterial suspensions were inoculated into MRS broth supplemented with 0.3%, 0.5%, or 1% (*w*/*v*) bile salts (oxgall; Sigma-Aldrich, St. Louis, MO, USA). Control cultures without bile salts were included. Cultures were incubated anaerobically at 37 °C for 24 h. Viable cell counts were determined at 0 and 24 h, and tolerance was evaluated based on survival relative to the control [15].

#### 2.4.3. Adhesion Ability to Intestinal Epithelium (Surface Hydrophobicity)

Cell surface hydrophobicity was evaluated using the microbial adhesion to hydrocarbons (MATH) method with xylene as the hydrophobic solvent. Xylene is commonly used in this method due to its nonpolar nature and its suitability for evaluating bacterial affinity for hydrophobic phases, which serves as a preliminary indicator of adhesion potential to intestinal epithelial surfaces. Bacterial suspensions were adjusted to an optical density (OD_600_) of 0.6 in PBS. After mixing with xylene and phase separation, OD_600_ values were measured, and hydrophobicity (%) was calculated.

%*H* = [(OD_600_ before mixing − OD_600_ after mixing)/OD_600_ before mixing] × 100

#### 2.4.4. Hemolytic Activity

Hemolytic activity was evaluated on tryptic soy agar (TSA) supplemented with 5% (*v*/*v*) defibrinated sheep blood (HiMedia Laboratories Pvt. Ltd., Mumbai, India). Plates were incubated at 37 °C for 48 h. Hemolysis patterns were classified as β-, α-, or γ-hemolysis based on visual inspection of the hemolytic zones surrounding colonies. *Staphylococcus aureus* ATCC 6538 was used as a positive control [16,17].

#### 2.4.5. Antimicrobial Activity Against *E. coli* and *S. aureus* ATCC 6538

Antibacterial activity was evaluated using the agar well diffusion method. Cell-free supernatants (CFS) were obtained by centrifugation and filtration (0.45 µm). Indicator strains included *Escherichia coli* and *Staphylococcus aureus* ATCC 6538. Wells were filled with 80 µL of CFS, and plates were incubated at 37 °C for 24 h. Lactic acid (2% *v*/*v*) was used as a positive control. Inhibition zones were measured in millimeters [18]. The pH of the cell-free supernatants was not neutralized or adjusted prior to testing; therefore, the observed inhibitory effects represent the combined activity of organic acids and other metabolites present in the supernatants, and should be interpreted as a preliminary assessment of antimicrobial potential rather than a specific bacteriocin effect.

### 2.5. Data Interpretation Probiotic Properties

#### 2.5.1. Acid Tolerance

Survival under acidic conditions was assessed by comparing bacterial counts (log CFU/mL) before and after incubation at pH 2.0 and pH 3.1. A reduction of less than 1 log CFU/mL at pH 3.1 was considered acceptable for acid tolerance [19].

#### 2.5.2. Bile Salt Tolerance

Bacterial counts (log CFU/mL) at 0 h and 24 h in MRS broth containing bile salts were compared. Strains were classified as tolerant if no marked reduction in viable counts was observed over the incubation period. The incubation period was applied as a stringent screening condition to assess bacterial survival under prolonged bile exposure rather than to directly simulate physiological intestinal transit time in piglets.

#### 2.5.3. Surface Adhesion Ability

Adhesion potential was expressed as surface hydrophobicity (%H) calculated by the MATH assay. Higher %H values indicated stronger cell surface adhesion capabilities. It should be noted that the MATH assay may be influenced by phase separation efficiency and the presence of emulsified droplets, which can affect OD_600_ measurements.

#### 2.5.4. Hemolytic Activity

Hemolysis was classified into three types: β-hemolysis, indicating complete lysis of red blood cells and the presence of a clear zone around the colony; α-hemolysis, representing partial lysis with a green or brown discoloration surrounding the colony; and γ-hemolysis, showing no lysis or visible change in the medium. According to safety criteria for probiotic use, LAB strains exhibiting α- or β-hemolysis were considered un-suitable, whereas only γ-hemolytic strains were deemed acceptable [20].

#### 2.5.5. Antimicrobial Activity Against *E. coli* and *S. aureus* ATCC 6538

The antimicrobial potential of the cell-free supernatants (CFS) was assessed by measuring the diameter of the inhibition zones against *Escherichia coli* and *Staphylococcus aureus* ATCC 6538. The *Escherichia coli* strain used in this study was a field isolate obtained from swine and maintained in the laboratory culture collection. The isolate was routinely cultured and characterized using standard microbiological methods prior to use in antimicrobial assays. The results were interpreted according to criteria adapted from Sirichokchatchawan et al. (2018) [18], as summarized in Table 1. This approach allowed for a comparative evaluation of the inhibitory effects of different CFS preparations on the selected pathogenic strains.

### 2.6. Molecular Identification of Selected Isolates

Selected isolates showing tolerance to acidic and bile conditions were identified using matrix-assisted laser desorption/ionization time-of-flight (MALDI-TOF) mass spectrometry (Bruker Daltonics GmbH, Bremen, Germany). MALDI-TOF MS is widely used as a rapid and reliable method for bacterial identification, including lactic acid bacteria, particularly in preliminary screening studies. Bacterial colonies were prepared according to the manufacturer’s instructions and analyzed using the reference database. Identification scores were interpreted as follows: ≥2.0 indicated reliable identification at the species level, 1.7–1.99 indicated genus-level identification, and <1.7 was considered unreliable. The closest matching species and corresponding score values were recorded for each isolate [13]. This approach was used for preliminary identification, and further molecular characterization (e.g., 16S rRNA sequencing) was considered beyond the scope of the present study.

### 2.7. Statistical Analysis

All experiments were performed in triplicate using the same bacterial isolate (technical replicates). Data were expressed as mean ± standard deviation (SD). As this study was designed as a preliminary in vitro screening without independent biological replication, inferential statistical analyses (e.g., *t*-tests or ANOVA) were not performed to avoid pseudo-replication and overinterpretation of the data. Instead, descriptive statistics were used to summarize the observed trends. This approach is consistent with previous screening-based probiotic studies. Statistical analysis was conducted using SPSS software (IBM SPSS Statistics, version 29).

## 3. Results

### 3.1. Isolation of Lactic Acid Bacteria (LAB) from Piglet Feces

From 42 fecal samples collected from suckling piglets, a total of 318 colonies exhibiting clear zones on MRS agar supplemented with CaCO_3_ were recovered after anaerobic incubation at 37 °C for 48 h. These colonies were considered presumptive lactic acid bacteria based on acid production. Gram staining identified 296 Gram-positive isolates, comprising 146 rod-shaped bacilli, 136 cocci, and 14 coccobacilli. Catalase testing indicated that 277 isolates were catalase-negative, whereas 19 catalase-positive isolates were excluded. Accordingly, 135 Gram-positive, rod-shaped, catalase-negative isolates were selected for further characterization.

**Figure 1:**
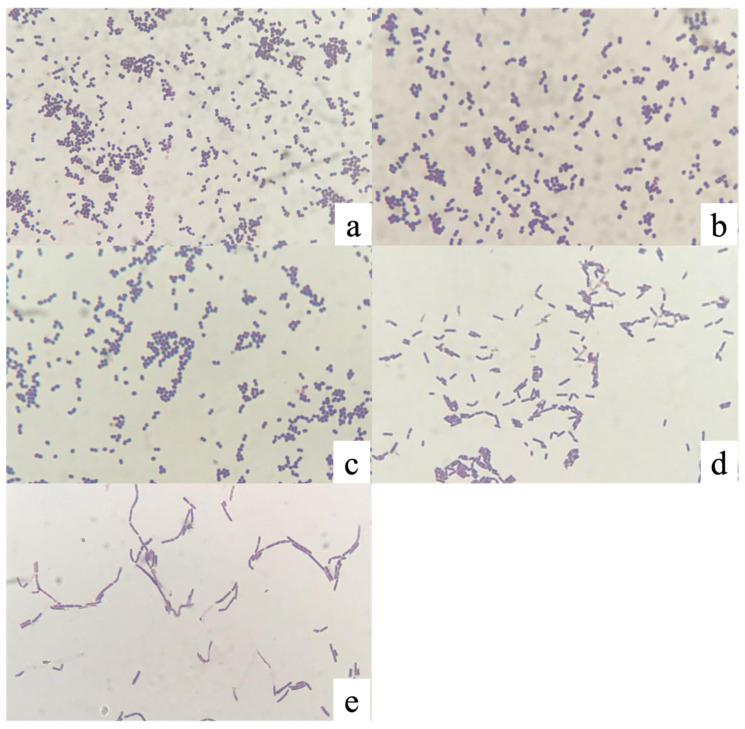
Microscopic images of representative isolates after Gram staining, observed under a light microscope at 1000× magnification: (**a**) PMvet120; (**b**) PMvet151; (**c**) PMvet183; (**d**) PMvet212; and (**e**) PMvet318.

Nineteen isolates exhibited budding and pleomorphic morphology consistent with yeast and were excluded from further analysis. In addition, three isolates were not maintained during subculturing. Among isolates with ambiguous Gram-staining results, PMvet120, PMvet151, and PMvet183 were confirmed as yeast-like organisms and excluded from the dataset (Figure 1).

### 3.2. Functional Properties of Selected Isolates

#### 3.2.1. Acid Tolerance

Among the 135 isolates screened, only two isolates (PMvet212 and PMvet318) demonstrated tolerance to acidic conditions. These isolates showed a reduction in viable counts of less than 1 log CFU/mL after incubation at pH 3.1 for 3 h. No isolates survived exposure to pH 2.0 under the same conditions. The survival profiles are summarized in Table 2.

#### 3.2.2. Bile Salt Tolerance

The two acid-tolerant isolates were further evaluated for bile salt tolerance. Both isolates maintained viable counts above 6 log CFU/mL after exposure to 0.3% (*w*/*v*) bile salts. At 0.5% bile salts, only PMvet212 retained substantial viability, whereas PMvet318 declined below the detection limit. No survival was observed for either isolate at 1.0% bile salts. The 0.3% (*w*/*v*) bile salt concentration was used to simulate physiological conditions of the small intestine, while 0.5% and 1.0% concentrations were applied to impose increasing selective pressure during in vitro screening. The survival data are presented in Table 3.

#### 3.2.3. Cell Surface Hydrophobicity (MATH Assay)

Cell surface hydrophobicity of the selected isolates showed inter-isolate variation. PMvet318 exhibited the highest hydrophobicity (12.38 ± 0.03%), followed by PMvet212 (7.85 ± 0.02%). These values indicate low to moderate adhesion potential based on the MATH assay. Variations in OD_600_ values, including occasional increases after mixing, may result from incomplete phase separation or transient emulsion formation inherent to the assay. Accordingly, measurements were recorded after phase stabilization, and results were interpreted as relative indicators of hydrophobicity. The results are summarized in Table 4.

#### 3.2.4. Hemolytic Activity (Safety Evaluation)

**Figure 2:**
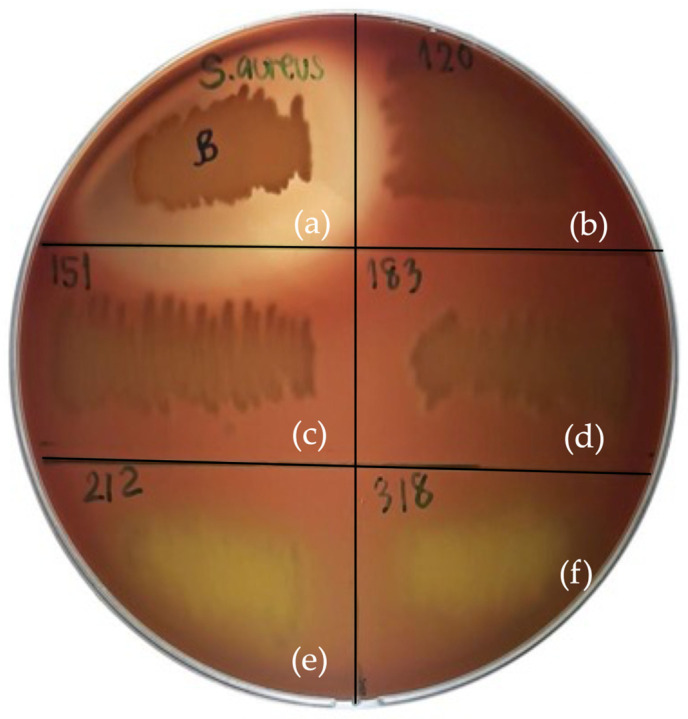
Hemolytic activity of selected LAB isolates on tryptic soy agar supplemented with 5% sheep blood after 48 h of incubation at 37 °C. (**a**) *Staphylococcus aureus* ATCC 6538 (positive control, β-hemolysis); (**b**) PMvet120, (**c**) PMvet151, and (**d**) PMvet183 showing γ-hemolysis (no lysis); (**e**) PMvet212 and (**f**) PMvet318 showing α-hemolysis (partial lysis, greenish discoloration).

Hemolytic activity of the selected isolates was assessed on blood agar. Both PMvet212 and PMvet318 exhibited α-hemolysis, characterized by greenish discoloration surrounding the colonies, indicating partial hemolysis (Figure 2). No β-hemolytic activity was observed. Based on established criteria for probiotic safety evaluation, the observed α-hemolysis suggests that these isolates may not be suitable candidates for probiotic application in their current form.

#### 3.2.5. Antimicrobial Activity Against *Escherichia coli* and *Staphylococcus aureus*

The inhibitory effects of the cell-free supernatants (CFS) against indicator pathogens were evaluated using the agar well diffusion assay against *Escherichia coli* and *Staphylococcus aureus*. PMvet212 and PMvet318 produced measurable inhibition zones against both target pathogens. As the cell-free supernatants were not pH-neutralized, the observed inhibitory activity likely reflects the combined effects of organic acid production and other metabolites. The results are presented in Table 5.

### 3.3. Molecular Identification of Selected Isolates

The two selected isolates were further identified using MALDI-TOF mass spectrometry. Isolate PMvet212 showed a high-confidence match with *Lactobacillus brevis* (score ≥2.0), indicating reliable identification at the species level. In contrast, PMvet318 yielded a lower score (<2.0) and was identified at the genus level as *Lactobacillus* sp. (Table 6). These results confirm that both isolates belong to the genus *Lactobacillus*, with one isolate identified at species level.

## 4. Discussion

The present study identified lactic acid bacteria (LAB) isolates from fecal samples of suckling piglets (7–28 days old) through a stepwise phenotypic screening approach. From an initial pool of 318 colonies, 135 Gram-positive, rod-shaped, catalase-negative isolates were retained for further evaluation. The detection of LAB in piglets at this early developmental stage is consistent with previous reports indicating that the gastrointestinal tract of piglets is rapidly colonized by *Lactobacillus* spp. prior to weaning [9,19]. This early colonization is influenced by maternal and environmental sources, including sow feces and milk, and contributes to the establishment of gut microbial stability and disease resistance [5,8,21]. The present findings support the concept that fecal samples from suckling piglets represent a relevant source of host-associated LAB for preliminary screening.

A high attrition rate was observed during the screening process. Although a large number of presumptive LAB isolates were initially obtained, only a small proportion demonstrated tolerance to gastrointestinal-like conditions, and none satisfied the predefined safety criteria due to α-hemolytic activity. These findings highlight the necessity of applying stringent multi-step selection criteria in the identification of probiotic candidates from natural sources.

Although yeast-like isolates were detected during the initial screening process, these were excluded from further analysis to maintain consistency in the evaluation of bacterial isolates. Previous studies have demonstrated that yeast species may exhibit probiotic-like properties, including resistance to gastrointestinal conditions and pathogen inhibition [22,23]. However, the present study was designed to focus specifically on LAB, and therefore yeast isolates were not included in subsequent analyses. Future studies may investigate these yeast isolates separately to assess their potential role in swine gut health.

The acid and bile tolerance assays revealed substantial variability among isolates. Of the 135 LAB isolates, only two (PMvet212 and PMvet318) demonstrated tolerance to acidic conditions (pH 3.1), whereas none survived at pH 2.0. This finding is consistent with previous reports indicating that tolerance to simulated gastric conditions is highly strain dependent [24]. Both isolates also exhibited measurable bile salt tolerance, with PMvet212 showing greater resistance at 0.5% bile concentration. Such tolerance is relevant for bacterial survival in the small intestine, where bile salts exert antimicrobial activity [25]. Given the preliminary in vitro screening design and use of technical replicates, these findings should be interpreted as indicative of stress tolerance rather than definitive evidence of gastrointestinal survival. The absence of survival at pH 2.0 likely reflects the stringent conditions of the assay rather than in vivo gastric environments, where shorter transit time and dietary buffering may mitigate acidity. Accordingly, the assay serves as a stress-based selection step within the screening pipeline. The observed strain-dependent survival pattern across increasing bile concentrations further supports the use of elevated bile levels as a selective screening rather than a physiological model. Moreover, it should be noted that this study was conducted using technical replicates without independent biological replication, which may limit the generalizability of the findings. Future studies should include biological replicates to improve robustness.

Molecular identification using MALDI-TOF mass spectrometry provided additional insight into the taxonomic identity of the selected isolates. PMvet212 was identified as *Lactobacillus brevis* with high confidence, whereas PMvet318 was identified at the genus level as *Lactobacillus* sp. The identification of *L. brevis* is consistent with previous reports describing its presence in the gastrointestinal tract of pigs and its potential role in producing antimicrobial metabolites and modulating gut microbiota [26]. The relatively lower identification score for PMvet318 suggests limitations in database matching or strain variability, highlighting the need for further molecular confirmation, such as 16S rRNA gene sequencing, to achieve precise taxonomic classification. The integration of MALDI-TOF identification strengthens the reliability of the present findings by linking phenotypic characteristics with taxonomic information [13].

In addition to phenotypic characterization, the present study employed MALDI-TOF mass spectrometry for the identification of selected isolates. This method provides a rapid and cost-effective protein fingerprinting approach for bacterial identification, enabling genus- and species-level classification within a short turnaround time [13]. However, compared with 16S rRNA gene sequencing, which is considered the reference method for resolving closely related species and confirming phylogenetic relationships, MALDI-TOF offers lower taxonomic resolution despite its practical advantages. Accordingly, MALDI-TOF is widely applied for preliminary identification in microbiological studies, whereas molecular approaches such as 16S rRNA gene sequencing are recommended for confirmatory analysis [27]. This limitation has become more evident following the taxonomic reclassification of the genus Lactobacillus in 2020, which reorganized this group into multiple novel genera within the family *Lactobacillaceae* [28]. As a result, the accuracy of MALDI-TOF MS identification may be affected, particularly when reference databases are not fully aligned with the revised classification system. Therefore, species-level identification based on MALDI-TOF should be interpreted with caution, and complementary molecular approaches, including 16S rRNA gene sequencing or whole-genome analysis, are necessary to ensure precise taxonomic classification and reliable biosafety evaluation. Previous studies have demonstrated that MALDI-TOF can reliably identify members of the genus *Lactobacillus*, including species commonly associated with the gastrointestinal tract of animals [13,29]. In swine, several LAB species, such as *Lactobacillus reuteri*, *Lactobacillus salivarius*, and *Lactobacillus plantarum*, have been frequently reported and are associated with gut microbial balance and host health [9,30], including pathogen inhibition, enhancement of intestinal barrier function, and modulation of immune responses.

The identification of isolates PMvet212 and PMvet318 as *Lactobacillus brevis* and *Lactobacillus* spp., respectively, using MALDI-TOF is consistent with previous reports describing *L. brevis* as a potential probiotic candidate in livestock and food systems. Several studies have shown that *L. brevis* exhibits desirable functional properties, including acid and bile tolerance, antimicrobial activity, and competitive exclusion of pathogens [31,32,33]. However, the present results indicate that probiotic potential is not universally associated with the genus, as α-hemolytic activity was observed in the selected isolates. This finding is consistent with increasing evidence that certain lactic acid bacteria may harbor genes associated with hemolytic or other virulence-related traits under specific conditions [34,35]. Accordingly, probiotic functionality should be considered strain-specific rather than species-dependent. These results further suggest that phenotypic screening alone is insufficient to confirm probiotic suitability, and that in vitro characteristics may not fully predict host performance. Therefore, in vivo validation is required to ensure both safety and functional efficacy of candidate probiotic strains.

Although the present study identified one isolate as *Lactobacillus brevis* and another at the genus level, these findings are consistent with the diversity of LAB populations in piglet gastrointestinal systems. Previous in vivo studies have shown that certain *Lactobacillus* species can improve growth performance, reduce diarrhea incidence, and enhance gut health in piglets [36,37]. However, it is important to emphasize that such effects are strain-specific and require comprehensive evaluation, including safety assessment and controlled in vivo trials. Therefore, while MALDI-TOF identification provides valuable preliminary taxonomic insight, further molecular confirmation and functional validation are necessary before translating these findings into practical applications.

Additional functional properties, including cell surface hydrophobicity and hemolytic activity, were also evaluated. Hydrophobicity values were relatively low (approximately 7–12%), suggesting limited adhesion potential based on the MATH assay alone. However, bacterial adhesion is a multifactorial process involving surface proteins, extracellular polysaccharides, and host interactions [38]. The observed variability and low hydrophobicity values may also reflect methodological limitations of the MATH assay, including sensitivity to phase separation and optical interference. In some cases, slight increases in OD_600_ after phase separation were observed, which may be attributed to light scattering caused by residual emulsified droplets or incomplete phase separation. Therefore, the hydrophobicity results should be interpreted with caution and considered as indicative rather than absolute measurements. Regarding safety, both PMvet212 and PMvet318 exhibited α-hemolysis, indicating partial hemolytic activity. Although α-hemolysis is less severe than β-hemolysis, current probiotic safety guidelines recommend the selection of non-hemolytic strains [10,39,40]. Accordingly, despite their functional characteristics, the isolates did not meet the required safety criteria for probiotic application. The presence of α-hemolysis may be associated with strain-specific traits or membrane-active compounds and warrants further genomic investigation. These findings emphasize that functional properties alone are insufficient for probiotic selection unless accompanied by rigorous safety confirmation. Therefore, integration of safety screening at early stages of candidate selection is essential, in line with established probiotic evaluation frameworks.

The antibacterial activity observed in PMvet212 and PMvet318 against *Escherichia coli* and *Staphylococcus aureus* suggests the production of inhibitory metabolites, such as organic acids or bacteriocin-like compounds [18,24]. The inhibition zones (10–12 mm) are consistent with values reported for LAB isolated from piglets [18], indicating moderate antibacterial activity. However, as the cell-free supernatants were not pH-neutralized prior to testing, the observed inhibition is likely primarily attributable to acid-mediated effects. Organic acid production is a key mechanism by which lactic acid bacteria inhibit enteric pathogens in vivo, particularly in early-life animals where gut pH plays an important role in microbial selection. Nonetheless, to differentiate between acid-dependent and acid-independent antimicrobial mechanisms, future studies should include pH-neutralized cell-free supernatant assays. Overall, this study provides preliminary screening data on LAB isolates from piglets and highlights the need for further molecular characterization, safety assessment, and in vivo validation to fully evaluate their functional potential.

## 5. Conclusions

This study provides a structured in vitro phenotypic screening of lactic acid bacteria isolated from feces of suckling piglets. From 318 initial colonies, 135 Gram-positive, rod-shaped, catalase-negative isolates were retained for evaluation, among which only two isolates (PMvet212 and PMvet318) demonstrated detectable tolerance to acidic and bile conditions, along with moderate antibacterial activity. The observed antimicrobial effects are likely associated with organic acid production and should be considered preliminary, requiring further validation using pH-controlled or pH-neutralized assays. Identification using MALDI-TOF mass spectrometry revealed that PMvet212 was closely related to *Lactobacillus brevis*, while PMvet318 was identified at the genus level as *Lactobacillus* spp. Further molecular identification using 16S rRNA sequencing and genome-based analyses is required to confirm taxonomic identity and ensure biosafety prior to any practical application. Despite these functional properties, both isolates exhibited α-hemolytic activity, indicating that they did not meet the safety criteria for probiotic application. Therefore, the findings of this study should be interpreted as preliminary evidence derived from an exploratory screening approach, rather than confirmation of probiotic suitability. Although selected isolates demonstrated characteristics associated with gastrointestinal stress tolerance and antimicrobial activity, additional investigations, including molecular confirmation, genome-based safety assessment, and controlled in vivo studies, are necessary before any practical application can be considered. Overall, this study provides a useful baseline for the identification and selection of host-associated LAB from piglets and supports future research aimed at developing evidence-based microbial strategies for improving gut health in swine production systems.

## Figures and Tables

**Table 1:** Interpretation of antimicrobial activity based on inhibition zone diameter.

Symbol	Interpretation	Inhibition Zone Diameter (mm)
−	Non-inhibition	<5
+	Weak inhibition	>5
++	Intermediate inhibition	>10
+++	Strong inhibition	>15
++++	Very strong inhibition	>20

**Table 2:** Survival of selected LAB isolates at pH 3.1 after 3 h of incubation at 37 °C. Values are expressed as mean ± standard deviation (n = 3).

Isolate ID	log CFU/mL	
	Initial Count	After Incubation at pH 3.1
120	6.42 ± 0.40	5.35 ± 0.40
151	6.30 ± 0.35	6.20 ± 0.30
183	6.40 ± 0.38	6.15 ± 0.70
212	6.37 ± 0.55	5.60 ± 0.50
318	6.37 ± 0.50	5.56 ± 0.60

All isolates were adjusted to similar initial concentrations prior to acid exposure.

**Table 3:** Survival of LAB isolates in MRS broth containing different bile salt concentrations after 24 h of incubation at 37 °C. Values are expressed as mean ± standard deviation (n = 3).

Isolate ID	0% (0 h)	0% (24 h, Control)	0.3% (24 h)	0.5% (24 h)
212	6.81 ± 0.4	9.66 ± 0.5	7.81 ± 0.3	6.56 ± 0.4
318	6.00 ± 0.6	9.08 ± 0.6	7.38 ± 0.5	<3.00

**Table 4:** Cell surface hydrophobicity (%) of selected LAB isolates as determined by the MATH assay using xylene. Values are expressed as mean ± standard deviation (n = 3).

Isolate ID	OD_600_ (Before Mixing with Xylene)	OD_600_ After Phase Separation, Aqueous Phase (0 Min)	OD_600_ After Phase Separation, Aqueous Phase (30 Min)	%Hydrophobicity (Mean ± SD)
212	0.871 ± 0.02	1.014 ± 0.02	0.924 ± 0.02	7.85 ± 0.02
318	0.935 ± 0.02	1.171 ± 0.03	1.010 ± 0.02	12.38 ± 0.03

**Table 5:** Antimicrobial activity of cell-free supernatants (CFS) from selected LAB isolates against *Escherichia coli* (field isolate) and *Staphylococcus aureus* ATCC 6538, determined by the agar well diffusion assay. Values are expressed as mean ± standard deviation (n = 3).

Isolate ID	Clear Zone (mm)—*E. coli*	Clear Zone (mm)—*S. aureus*
PMvet212	12.50 ± 0.4	11.75 ± 0.5
PMvet318	11.50 ± 0.3	10.50 ± 0.4
Control (2% lactic acid)	18.50 ± 0.5	15.25 ± 0.4

**Table 6:** Identification of selected LAB isolates using MALDI-TOF.

Isolate ID	Closest Match	Score Value	Identification Level
PMvet212	*Lactobacillus brevis*	2.1–2.3	Species level
PMvet318	*Lactobacillus* sp.	1.7–1.9	Genus level

## Data Availability

Relevant information is included in the article.
